# The effects of mindfulness-based interventions on anxiety, depression, stress, and mindfulness in menopausal women: A systematic review and meta-analysis

**DOI:** 10.3389/fpubh.2022.1045642

**Published:** 2023-01-09

**Authors:** Hongyang Liu, Kexin Cai, Jinyang Wang, Hailian Zhang

**Affiliations:** School of Nursing, Yanbian University, Yanji, China

**Keywords:** mindfulness, menopausal, anxiety, depression, stress, meta-analysis

## Abstract

**Background:**

Mindfulness-based interventions (MBIs) are psychological interventions widely used in menopausal women. Currently, there is no evidence summary on the effectiveness of MBIs on anxiety, depression, stress, and mindfulness in menopausal women. This meta-analysis examines the effectiveness of MBIs in improving anxiety, depression, stress, and mindfulness scores in menopausal women.

**Methods:**

A systematic search was conducted in PubMed, Embase, Web of Science, the Cochrane Library, CNKI (China National Knowledge Infrastructure), and Wanfang, using relevant terms such as MBIs as keywords and covering all studies published before March 13, 2022. The outcomes were anxiety, depression, stress, and mindfulness. The screening and extraction of data were conducted by two independent reviewers.

**Results:**

A total of 1,138 menopausal women participated in 13 studies. Meta-analysis results showed that MBIs significantly reduced stress in menopausal women (SMD = −0.84, 95% CI: −1.64 to −0.05, *p* = 0.04), but no statistical differences were found in reducing anxiety (SMD = −0.40, 95% CI: −0.81 to 0.01, *p* = 0.06) and depression (SMD = −0.19, 95% CI: −0.45 to 0.07, *p* = 0.16) and in raising the scores of mindfulness (SMD = 0.37, 95% CI: −0.06 to 0.81, *p* = 0.09) in menopausal women.

**Conclusion:**

MBIs may reduce stress in menopausal women, but their effect on improving anxiety, depression, and mindfulness needs further validation.

**Systematic review registration:**

https://www.crd.york.ac.uk/prospero/#recordDetails.

## 1. Introduction

Menopause refers to the decline of ovarian function and the cessation of menstruation (1). During the menopause, women have a series of neuropsychological symptoms, mainly the dysfunction of the autonomic nervous system, which is caused by the fluctuation or decrease of sex hormones (2).

Studies showed that the probability of anxiety and depression in menopausal women is 12.62 and 25.99%, respectively, due to the variability and complexity of emotions at this stage. The risk can be three times greater than it was before menopause (3, 4). Adverse psychological emotions will reflect the functions of body organs and systems through immune and endocrine mechanisms, which directly affect the physical and mental health of menopausal women (5). Furthermore, this will expose menopausal women to enormous psychological and social challenges, which can seriously affect their quality of life in turn (6). Therefore, scholars are actively exploring scientific and effective interventions to improve negative emotions and cope with stress in menopausal women. According to the 2018 Guideline for Evaluation and Treatment of Menopausal Depression (7), psychological interventions or pharmacotherapy could be used as the first-line treatment for anxiety and depression in menopausal women. In contrast, psychological interventions have fewer adverse effects and better long-term results than pharmacotherapy (8), which most importantly meets the willingness of 80% of women to use them (9–11). Mindfulness-based interventions (MBIs) have been shown to effectively alleviate negative emotions such as anxiety, depression, and stress as one of the psychological interventions. Moreover, MBIs are also supposed to have promising therapeutic effects on mental and chronic diseases (12–14).

Mindfulness implies that participants establish a new perspective on themselves, consciously focus on the goal of the present moment, and approach the various experiences unfolding in the present moment without judgment (15, 16). These experiences can take many forms, such as personal physical sensations, emotional reactions, mental pictures, mental conversations, and perceptual experiences (17). Historically, mindfulness, known as the “heart” of Buddhist meditation (18, 19), originated in Buddhism. Buddhist culture, therefore, provides a wealth of information for the psychological study of mindfulness, but mindfulness is by no means Buddhism or Buddhist meditation practices. MBIs are an umbrella term for a range of “mindfulness”-centered, de-religious psychological interventions, such as mindfulness-based stress reduction therapy (MBSR) (15), mindfulness-based cognitive therapy (MBCT) (20), and brief mindfulness meditation training. However, there are also many mindfulness-related interventions that incorporate mindfulness training as an integral part of a comprehensive treatment program, such as dialectical behavior therapy (DBT) (21, 22), acceptance and commitment therapy (ACT) (23), and integrated mind-body training (24). Its basic mechanism is to focus attention on the present moment with a nonjudgmental attitude and to disengage oneself from wandering, triggering the experience of re-perception and thus self-emotional regulation, which helps reduce negative emotions more effectively (25). The purpose of MBIs is to facilitate the opening of one's thoughts and feelings when one is in an anxious or depressive thinking pattern and bodily experience, which helps to reduce anxiety and depression triggered avoidance, rumors, and self-judgment through the process of attention and consciousness turning (26). Furthermore, MBIs can foster greater awareness of inner body feelings and emotional regulation, promote stress resilience, and improve stress management and stress coping skills, which ultimately help alleviate anxiety, depression, and stress in menopausal women (17).

Currently, there is an increase in the number of RCTs on the use of MBIs in menopausal women. There are more empirical studies analyzing the effectiveness of MBIs among menopausal women. However, there is no consistent data on its effectiveness in improving anxiety, depression, stress, and mindfulness in menopausal women. Several studies have shown that MBIs can significantly reduce anxiety, depression, and stress scores (27–35), while some studies did not have statistically significant results (9, 36). Some studies have shown that interventions significantly enhanced mindfulness scores (33, 37, 38), while other studies showed no significant effect of interventions on mindfulness scores (9). Current analyses are controversial about the effectiveness of MBIs for menopausal women to improve anxiety, depression, stress, and mindfulness. These controversies need to be further clarified through a systematic integration of the available evidence. Therefore, this study systematically searched and reviewed the evidence on the effectiveness of MBIs for improving anxiety, depression, stress, and mindfulness in menopausal women, conducting a meta-analysis of existing studies in the context of global trends in integration.

This study aims at informing the implementation of more effective and sustainable community-based MBIs in different cultural contexts by providing evidence-based support for the development of interventions to improve anxiety, depression, stress, and mindfulness in menopausal women. Public health agencies, therefore, can clearly understand current effective interventions in mental health training for menopause care. The next step is to determine what measures should be taken to achieve widespread training implementation.

## 2. Materials and methods

The PRISMA Guidelines (39) and the Cochrane Handbook of Systematic Review (40) were used to do a systematic review and meta-analysis of this study. We were registered in the PROSPERO Registry (CRD42022319349).

### 2.1. Search strategy

Two reviewers (HL and JW) independently searched the following databases: PubMed, the Cochrane Library, Embase, Web of Science, CNKI (China National Knowledge Infrastructure), and Wanfang in order to achieve a more systematic retrieval, covering all studies published before March 13, 2022. The search strategy should be as comprehensive as possible but also be modified according to the requirements of different databases (Supplementary material). Also, the available references were further filtered by searching for relevant reviews, meta-analyses, or systematic reviews. This made sure that the search was as complete as possible.

### 2.2. Inclusion criteria and exclusion criteria

The inclusion criteria of this study were formulated according to the PICOS principles as follows: (1) P: The subjects met the diagnostic criteria for menopause, and their age was not limited. (2) I: The experimental group needs to use MBIs for menopausal women (e.g., MBSR, MBCT, DBT, ACT, mindfulness yoga, mindfulness meditation, and so on, without limitation on intervention time). C: The control group required a different intervention (e.g., wait-list, routine health care, general conversation, and so on). (4) O: The outcomes were anxiety, depression, stress, or mindfulness in menopausal women (without specific outcome measures specified). (5) S: The study type was RCTs. Exclusion criteria are as follows: (1) repeated publication; (2) inability to obtain the full text; (3) incomplete or unavailable data; (4) studies that have not been published in Chinese or English.

### 2.3. Data extraction

After duplicate studies were removed (EndNote X9), titles and abstracts were screened by two reviewers (KC and JW) independently. All potentially eligible studies were independently evaluated for the full text based on inclusion and exclusion criteria. Any disagreements were resolved in consultation with the third reviewer (KC). The information extracted from the included articles contained: the author (year), country, participants, sample size (E/C), intervention (E/C), length of intervention (weeks), and outcomes (instrument).

### 2.4. Quality assessment

The RCT bias risk assessment tool recommended by the Cochrane Systematic Review Manual (5.1.0) (40) was used to strictly evaluate the quality of the included literature. The degree of risk of bias for each included article will be assessed as “low risk,” “unclear” or “high risk,” to be completed independently by two reviewers (KC and JW). Review of the final results and resolving disagreements will be done by the third reviewer (HL).

### 2.5. Statistical analysis

Statistical analysis was conducted under the guidance of the corresponding author (HL), a statistics expert. All reviewers are aware of the statistical analyses currently being carried out. Statistical analysis was performed using RevMan 5.4. Statistical heterogeneity between studies will be analyzed by the chi-square test and the I^2^ statistic (41) before results are integrated. If *p* ≥ 0. 10 and I^2^ <50% indicate low heterogeneity, the fixed-effects model is used. The fixed-effects model is used if *p* ≥ 0.10 and I^2^ <50% indicate low heterogeneity. A random-effects model was chosen if *p* < 0.10 and I^2^ ≥ 50%, indicating high heterogeneity, and possible sources of heterogeneity were investigated using subgroup or sensitivity analysis (42). There may be potential confounding factors affecting the intervention effect and the risk of heterogeneity (43) due to the large and differentiated sample sizes of the included studies and the failure to unify the intervention methods, and a random-effects model was used to integrate (44). We used the standard mean difference (SMD) in this study, and the 95% CI was used to indicate the summary result, in which case it is necessary to mark the study's results as a unified measure unit (40). MBIs were considered statistically significant in the overall effect if *p* < 0.05, and vice versa. The clinical significance of SMD was evaluated by Marfo's explanation of effect size (low, medium, and high were respectively <0.40, 0.40–0.70, and >0.70) (45). If a meta-analysis includes ≥10 studies, publication bias needs to be assessed by a funnel plot (46).

### 2.6. Subgroup analyses

Subgroup analyses were conducted to explore the following moderators: continent and length of intervention (weeks).

## 3. Results

### 3.1. Study selection

**Figure 1:**
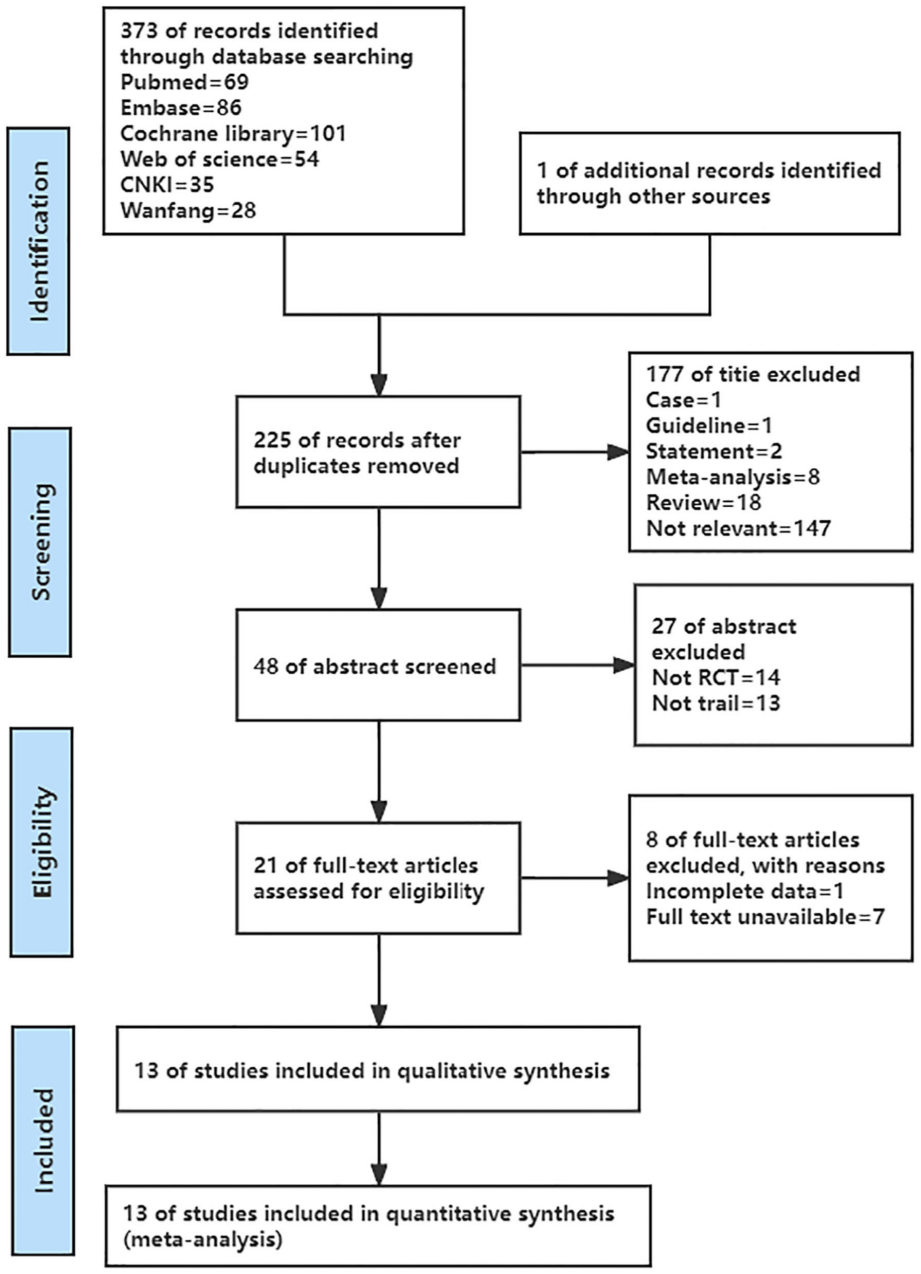
PRISMA flow chart of study selection.

The details of the literature screening process are presented in Figure 1. 374 English and Chinese articles were retrieved initially, and 225 articles were extracted after 149 duplicates were removed. After reading the titles and abstracts, eliminate 204 articles, leaving 21 articles. After reading the full text, 13 articles met the inclusion criteria. As a result, the final 13 articles were included in this meta-analysis (9, 27–38).

### 3.2. Characteristics of articles

The general characteristics of the included studies are listed in Table 1. A total of 13 studies published before 2022 were included. Sample sizes for each study ranged from 27 to 197, and 1,138 menopausal women aged 40–70 years were recruited for all included studies, including 560 participants in the experimental group and 578 participants in the control group. The participants were menopausal women with menopausal symptoms who had not been diagnosed with a psychiatric disorder. According to the modes of menopause, there are natural menopause (9, 28, 38), natural menopause, and iatrogenic menopause (27, 29–37). Three studies (28, 37, 38) included women over 1 year after menopause. Four studies (9, 33, 34, 36) excluded women who had previously participated in formal MBIs. Seven studies (9, 29–32, 34, 38) excluded menopausal women treated with hormones. The interventions were all based on mindfulness and ranged in duration from 8 to 12 weeks, with interventions ranging from 0.5 to 5 hours per week. Except for Fu-Zhen Zhong (27), Chattha et al. (34), and Monfaredi et al. (35), all experimental groups adopted the method of group training combined with individual training. Four studies (29–32) further subdivided training methods into formal and informal training methods. Formal training methods include body scanning, mindful yoga, sitting meditation, mindful walking, etc. Informal methods include detecting pleasant and unpleasant events, detecting breathing, eating, walking, and other daily activities. The control group received wait-list (27, 29, 31–33, 36, 38), menopause hormone therapy (28), routine health care (30, 35), menopause education control (9), and easy body movements (34). Outcome indicators included anxiety, depression, stress, and mindfulness scores.

**Table 1:** Characteristics of the included studies.

**Author (year)**	**Country**	**Participants**	**Sample size**	**Intervention**	**length of intervention (weeks)**	**Outcomes (instrument)**
			**E/C**	**E/C**		
Fu-Zhen Zhong et al. (27)	China	Menopausal women	13/14	MT/WL	8 weeks	Anxiety: STAI Depression: SDS
Fen-Xia (28)	China	Menopausal women	35/35	MHT+MBSR/ MHT	8 weeks (2 h per week)	Anxiety: GAD-7 Depression: PHQ-9
Fu-Zhen Zhong et al. (29)	China	Menopausal women	36/37	MT/WL	8 weeks (2.5 h per week)	Anxiety: bPOMS-Anxiety Depression: bPOMS-Depression
Hong-Yan Cheng and Xiao-Yan (30)	China	Menopausal women	80/80	MT/ RHC	8 weeks (2.5 h per week)	Anxiety: SAS Depression: SDS
Wen Xu et al. (31)	China	Menopausal women	35/39	MT/WL	8 weeks (2.5 h per week)	Depression: SDS
Shu-Xia Wang et al. (32)	China	Menopausal women	29/31	MT/WL	8 weeks (2.5 h per week)	Depression: SDS
Garcia et al. (38)	Brazil	Postmenopausal women	19/11	MT/GC	8 weeks (0.5 h per week)	Mindfulness: MAAS
Sener et al. (38)	Turkey	Postmenopausal women	55/63	MBSR/WL	16 weeks (2.5 h per week)	Mindfulness: MAAS
Gordon et al. (33)	Canada	Menopause transition women	44/51	MBSR/ WL	8 weeks (2.5 h per week)	Anxiety: STAI Depression: CES-D Stress: PSS Mindfulness: FFMQ
Wong et al. (9)	Hong Kong, China	Peri-menopausal or post-menopausal women	98/99	MBSR/MEC	8 weeks (2.5 h per week)	Stress: PSS Mindfulness: FFMQ
Chattha et al. (34)	India	Menopausal women	54/54	IAYT/ EBM	8 weeks (5 h per week)	Stress: PSS
Cramer et al. (36)	Germany	Menopausal women	19/21	YM/WL	12 weeks (1.5 h per week)	Anxiety: HADS-Anxiety Depression: HADS-Depression
Monfaredi et al. (35)	Iran	Postmenopausal women	43/43	ACT/ RHC	8 weeks (1-1.5 h per week)	Anxiety: DASS 21-Anxiety Depression: DASS 21-Depression Stress: DASS 21-Stress

E, experimental group; C, control group; MT, mindfulness training; WL, wait-list; MHT, menopause hormone therapy; MBSR, mindfulness-based stress reduction; RHC, routine health care; GC, general conversation; MEC, menopause education control; IAYT, integrated approach to yoga therapy; EBM, easy body movements; YM, yoga and meditation; ACT, acceptance and commitment training; STAI, State-Trait Anxiety Inventory; GAD-7, Generalized Anxiety Disease-7; SAS, Self-rating Anxiety Scale; HADS, Hospital Anxiety and Depression Scale; DASS 21, Depression, Anxiety, Stress Scale-21; bPOMS, brief Profile of Mood States; SDS, Self-rating Depression Scale; PHQ-9, Patient Health Questionnaire-9; CES-D, Center for Epidemiologic Studies Depression Scale; PSS, Perceived Stress Scale; MAAS, Mindful Attention Awareness Scale; FFMQ, Five Facet Mindfulness Questionnaire.

### 3.3. Risk of bias in the included literature

**Figure 2:**
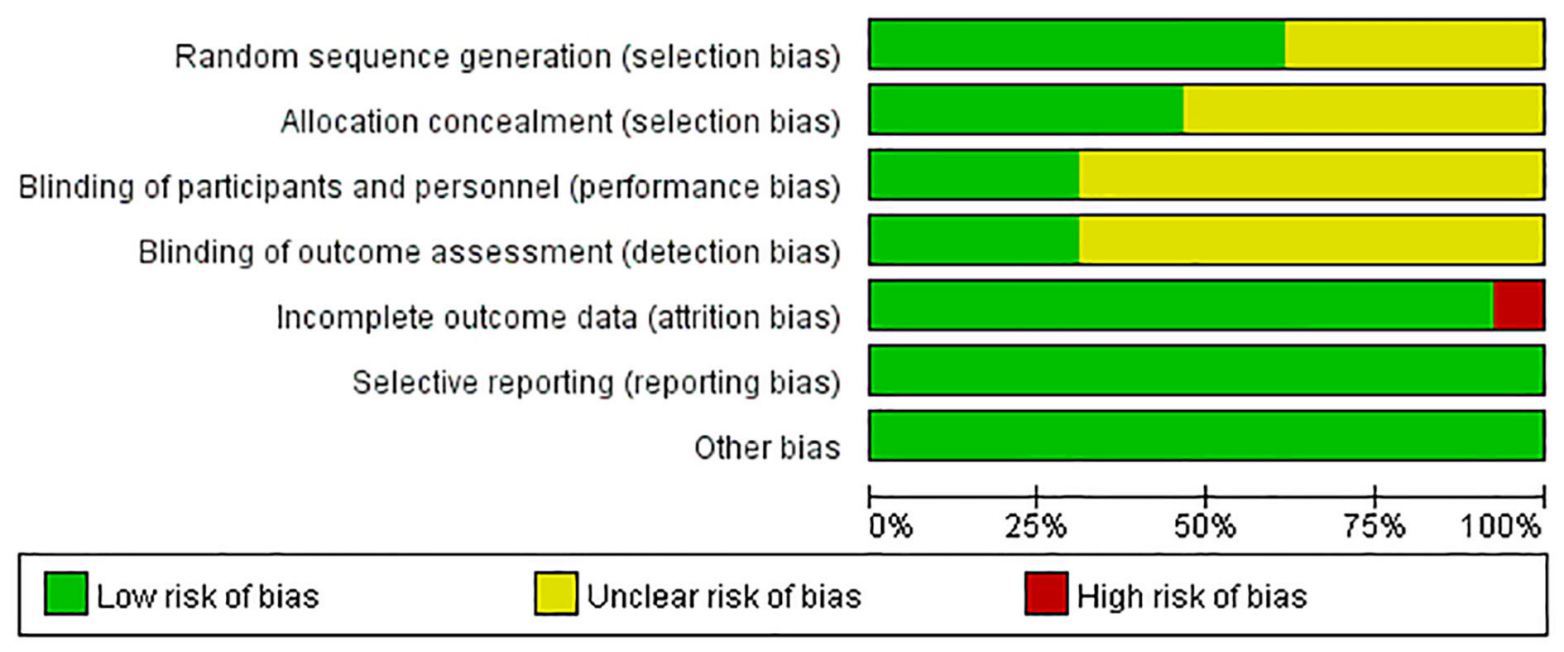
Risk of bias graph.

Of these 13 studies, eight used an appropriate sequence generation process, six had adequate concealment of allocation, four used blinding of participants and performers, four implemented blinding of outcome assessments, 12 ensured the completeness of outcome data, and 13 had selective reporting of low risk of bias. Specific information is detailed in Table 2, and the results of the risk bias assessment are provided in Figure 2. Among the 13 RCTs included, participants were randomly grouped in all the included studies, but only the specific random allocation sequence generation method was described in five studies (9, 33–36) that employed computer software; two studies (28, 30) used a random number table; and one study (37) used the random drawing method for random grouping. Three studies (33, 35, 36) used sealed and opaque envelopes with serial numbers; one study (37) used boxes; and one study (34) used a central random allocation system for hiding distribution. In Wong's study (9), a statistician who was not part of the research team performed random number generation and allocation. Participants were unaware of the results of randomization when they filled out the baseline questionnaire. Blinding is difficult because of the nature of intervention studies. Wong et al. (9) adopted a single-blind design with participant blindness. Nurdilan et al. (38) prevented data contamination between groups by collecting experimental and control data in different health centers. Gordon et al. (33) gave participants instructions for the next step through email; Chattha et al. (34) employed a blind method to conduct random assignment and statistical analysis, and the survey questionnaire was coded and decrypted after the analysis was completed. The class time and place of the experimental group and the control group were reasonably arranged to avoid interaction and communication between the participants of the two groups. All the studies described the number of cases lost to follow-up during the study period and the reasons for the loss, but the missing rate of Nurdilan et al. (38) was > 20%, leading to a high risk of bias. In all studies and all reported study regimens, there were no statistically significant differences at baseline between the experimental and control groups.

**Table 2:** Literature quality assessment.

**Author (year)**	**Random sequence generation**	**Allocation concealment**	**Blind method**	**Outcome data**	**Selective reporting**	**Other bias**	**Literature quality**
Fu-Zhen Zhong et al. (27)	Unclear	Unclear	Unclear	Low-risk bias	Low-risk bias	Low-risk bias	B
Fen-Xia (28)	Low-risk bias	Unclear	Unclear	Low-risk bias	Low-risk bias	Low-risk bias	B
Fu-Zhen Zhong et al. (29)	Unclear	Unclear	Unclear	Low-risk bias	Low-risk bias	Low-risk bias	B
Hong-Yan Cheng and Xiao-Yan (30)	Low-risk bias	Unclear	Unclear	Low-risk bias	Low-risk bias	Low-risk bias	B
Wen Xu et al. (31)	Unclear	Unclear	Unclear	Low-risk bias	Low-risk bias	Low-risk bias	B
Shu-Xia Wang et al. (32)	Unclear	Unclear	Unclear	Low-risk bias	Low-risk bias	Low-risk bias	B
Garcia et al. (38)	Low-risk bias	Low-risk bias	Unclear	Low-risk bias	Low-risk bias	Low-risk bias	B
Sener et al. (38)	Unclear	Unclear	Low-risk bias	High-risk bias	Low-risk bias	Low-risk bias	B
Gordon et al. (33)	Low-risk bias	Low-risk bias	Low-risk bias	Low-risk bias	Low-risk bias	Low-risk bias	A
Wong et al. (9)	Low-risk bias	Low-risk bias	Low-risk bias	Low-risk bias	Low-risk bias	Low-risk bias	A
Chattha et al. (34)	Low-risk bias	Low-risk bias	Low-risk bias	Low-risk bias	Low-risk bias	Low-risk bias	A
Cramer et al. (36)	Low-risk bias	Low-risk bias	Unclear	Low-risk bias	Low-risk bias	Low-risk bias	B
Monfaredi et al. (35)	Low-risk bias	Low-risk bias	Unclear	Low-risk bias	Low-risk bias	Low-risk bias	B

### 3.4. Meta-analysis results

#### 3.4.1. Anxiety scores

**Figure 3:**
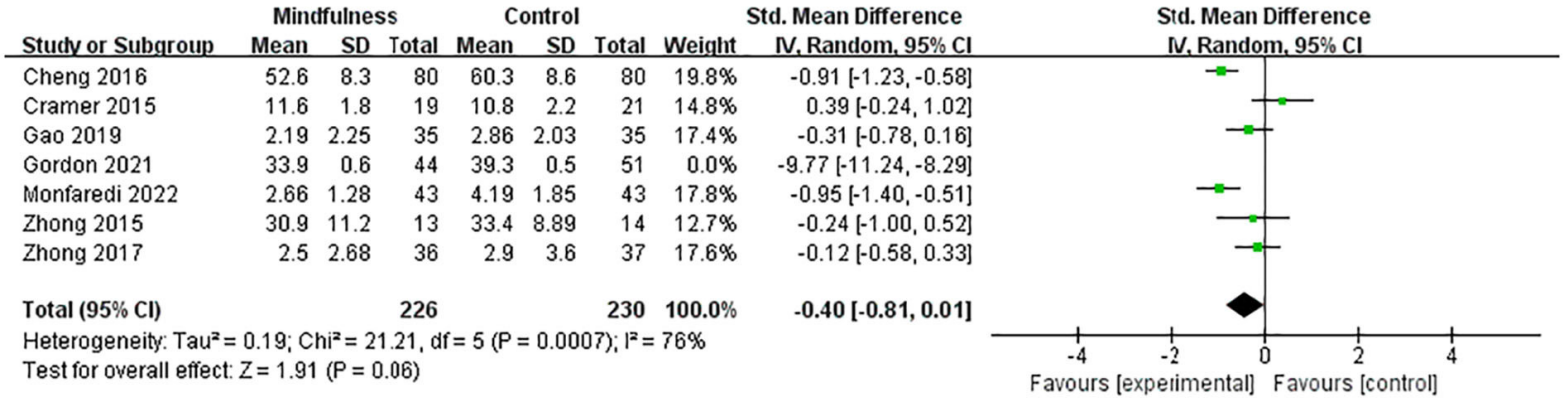
Forest plot of the anxiety scores.

Seven existing studies (27–30, 33, 35, 36) recruited 551 menopausal women (270 in the experimental group and 281 in the control group) to evaluate the effects of MBIs on anxiety scores in menopausal women using the STAI, GAD-7, SAS, HADS, DASS 21, and bPOMS, respectively. SMD was used to deal with numerical variables due to different evaluation tools. The heterogeneity test showed significant heterogeneity among studies (*p* < 0.01, I^2^ = 96%). Thus, a random-effects model was used. The results showed that the experimental group significantly reduced anxiety scores in menopausal women compared to the control group (SMD = −1.47, 95% CI: −2.52 to −0.42, *p* < 0.01), with a high effect size. A sensitivity analysis was conducted to investigate the impact of each study by removing one study at a time. Sensitivity analysis showed an SMD range from −1.80 (95% CI: −2.97 to −0.62) to −0.40 (95% CI: −0.81 to 0.01) for each combination. Gordon's study (33) had the largest effect on the combined effect size. The results showed that after removing the maximum outlier (I^2^ = 76%), the anxiety scores of the experimental group were lower than those of the control group. The difference between the two groups was not statistically significant (SMD = −0.40, 95% CI: −0.81 to 0.01, *p* = 0.06) (Figure 3), indicating that the MBIs had no significant effect on anxiety in menopausal women.

#### 3.4.2. Depression scores

**Figure 4:**
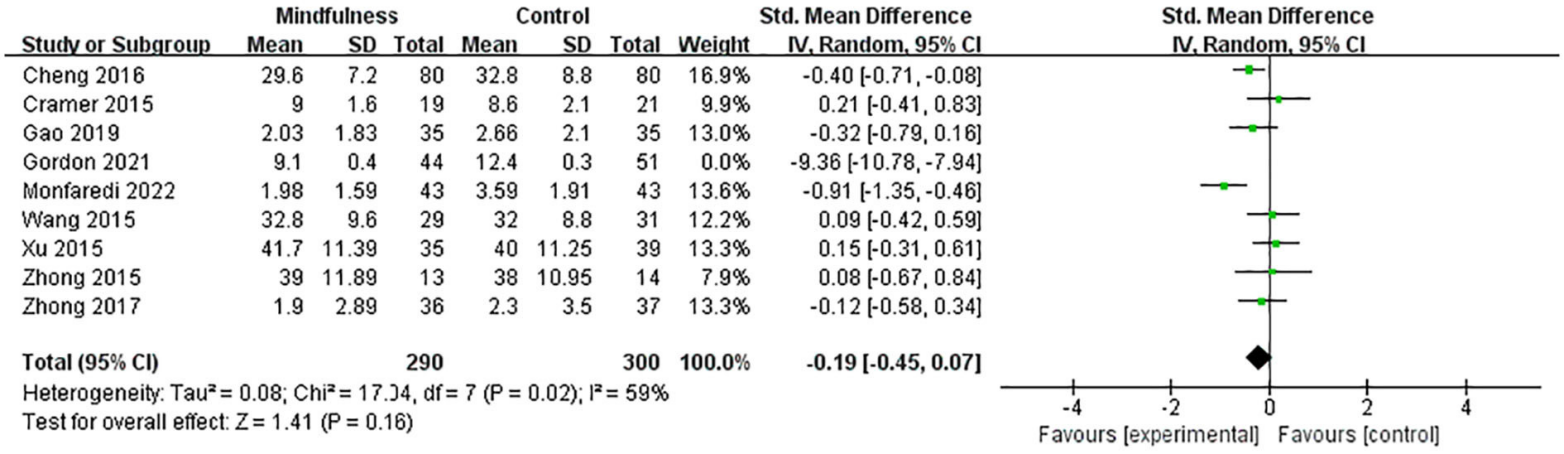
Forest plot of the depression scores.

Nine studies (27–33, 35, 36) included 685 menopausal women (334 in the experimental group and 351 in the control group) to evaluate the effects of MBIs on depression scores in menopausal women using the SDS, PHQ-9, HADS, CES-D, DASS 21, and bPOMS, respectively. SMD was used to deal with numerical variables due to different evaluation scales. The heterogeneity test showed significant heterogeneity among studies (*p* < 0.01, I^2^ = 95%). Thus, a random-effects model was adopted. The results showed that compared with the control group, the experimental group significantly reduced the depression score of menopausal women (SMD = −0.95, 95% CI: −1.74 to −0.16, *p* = 0.02), with a high effect size. A sensitivity analysis was performed to investigate the impact of each study by deleting one study at a time. A sensitivity analysis showed an SMD range from −1.12 (95% CI: −2.01 to −0.23) to −0.19 (95% CI: −0.45 to 0.07) for each combination, with Gordon's study (33) being the study that had the most significant impact on the combined effect size. The results showed that after removing the maximum outlier (I^2^ = 59%), the depression scores of the experimental group were lower than those of the control group. There was no statistically significant difference between the two groups (SMD = −0.19, 95% CI: −0.45 to 0.07, *p* = 0.16) (Figure 4), indicating that the MBIs had no significant effect on depression in menopausal women.

#### 3.4.3. Stress scores

**Figure 5:**
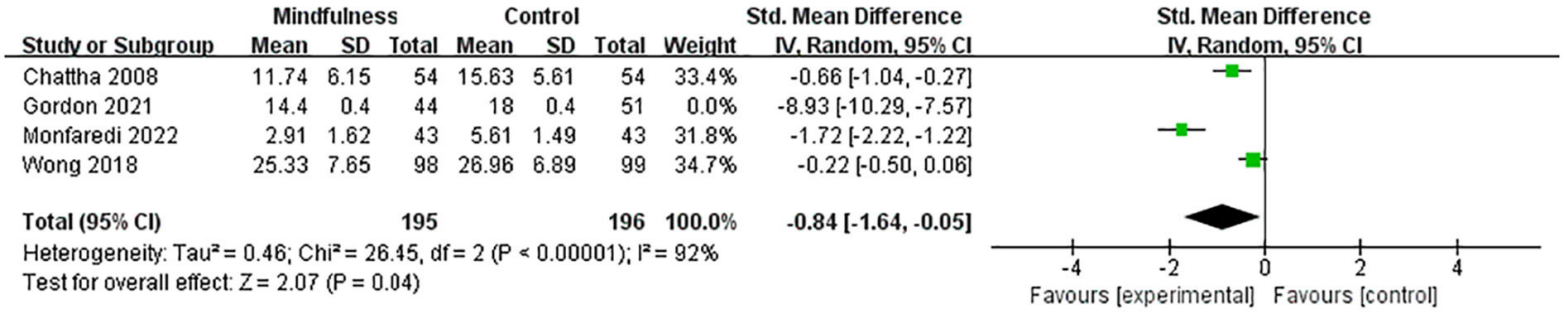
Forest plot of the stress scores.

Four existing studies (9, 33–35) recruited 486 menopausal women (239 in the experimental group and 247 in the control group) to evaluate the effects of MBIs on stress scores in menopausal women using the PSS and DASS 21, respectively. Since the evaluation instruments were different, the numerical variables were treated with SMD. The heterogeneity test showed significant heterogeneity among studies (*p* < 0.01, I^2^ = 98%). Thus, a random-effects model was used. The results showed that the experimental group significantly reduced anxiety scores in menopausal women compared to the control group (SMD = −2.68, 95% CI: −4.39 to −0.96, *p* = 0.002), with a high effect size. A sensitivity analysis was conducted to investigate the impact of each study by removing one study at a time. Sensitivity analysis showed an SMD range from −3.65 (95% CI: −6.44 to −0.86) to −0.84 (95% CI: −1.64 to −0.05) for each combination. Gordon's study (33) had the largest effect on the combined effect size. The results showed that after removing the maximum outlier (I^2^ = 92%), the anxiety scores of the experimental group were lower than those of the control group. And the difference between the two groups was statistically significant (SMD = −0.84, 95% CI: −1.64 to −0.05, *p* = 0.04) (Figure 5), indicating that the MBIs had a significant effect on stress in menopausal women. The result did not change when combined using the fixed-effects model (SMD = −0.60, 95% CI: −0.81 to −0.40, *p* < 0.01), indicating that the result of this meta-analysis was robust.

#### 3.4.4. Mindfulness scores

**Figure 6:**
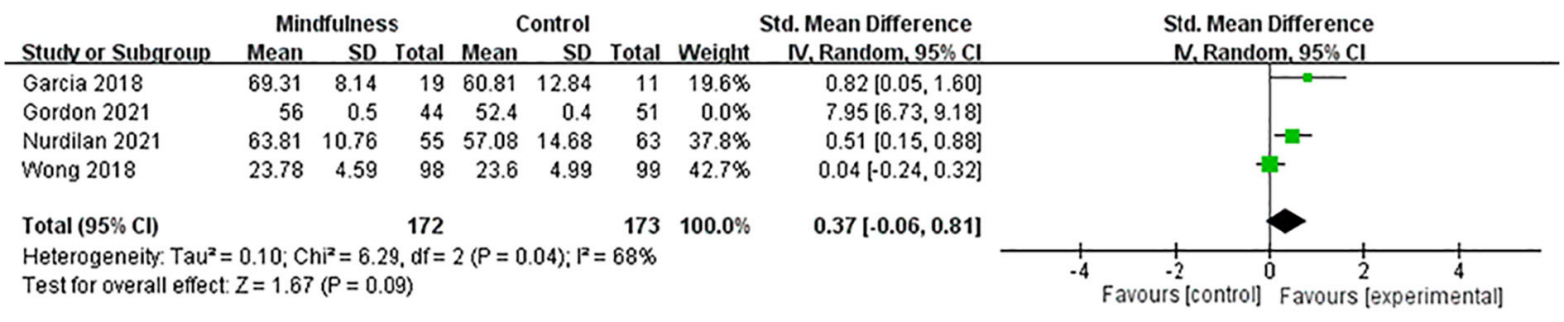
Forest plot of the mindfulness scores.

Four studies (9, 33, 37, 38) included 440 menopausal women (216 in the experimental group and 224 in the control group) to evaluate the effects of MBIs on mindfulness scores in menopausal women using the MAAS and FFMQ, respectively. SMD was used to deal with numerical variables due to different evaluation scales. The heterogeneity test showed significant heterogeneity among studies (*p* < 0.01, I^2^ = 98%). Thus, a random-effects model was adopted. The results showed that compared with the control group, the experimental group significantly increased the mindfulness scores of menopausal women (SMD = 2.20, 95% CI: 0.43 to 3.96, *p* = 0.01), with a high effect size. The sensitivity analysis was performed to investigate the impact of each study by deleting one study at a time. The sensitivity analysis showed an SMD range from 0.37 (95% CI: −0.06 to 0.81) to 3.04 (95% CI: −0.37 to 6.45) for each combination, with Gordon's study (33) being the study that had the greatest impact on the combined effect size. The results showed that after removing the maximum outlier (I^2^ = 68%), the mindfulness scores of the experimental group were higher than those of the control group. There was no statistically significant difference between the two groups (SMD = 0.37, 95% CI: −0.06 to 0.81, *p* = 0.09) (Figure 6), indicating that the MBIs had no significant effect on mindfulness scores in menopausal women.

### 3.5. Subgroup analyses

Subgroup analyses were conducted on the continent and length of intervention (weeks) for the outcomes—anxiety, depression, and mindfulness.

#### 3.5.1. Continent

For anxiety, there were significant differences in SMD between the two subgroups: Asian (27–30, 35) and Europe (36) (*p* = 0.01). MBIs had significant effects among the study with Asian (SMD = −0.55, 95% CI: −0.91 to −0.18, *p* = 0.003). However, no significant intervention effect was found for Europe (SMD = 0.39, 95% CI: −0.24 to 1.02, *p* = 0.23). For depression, there were no significant differences in SMD between the two subgroups: Asian (27–32, 35) and Europe (36) (*p* = 0.20). MBIs did not find a significant intervention effect in Asian (SMD = −0.23, 95% CI: −0.51 to 0.04, *p* = 0.10) and Europe (SMD = 0.21, 95% CI: −0.41 to 0.83, *p* = 0.51).

#### 3.5.2. Length of intervention (weeks)

For depression, there were no significant differences in SMD between the two subgroups: 8 weeks (27–33, 35), 12 weeks (36) (*p* = 0.20). MBIs did not find a significant intervention effect at 8 weeks (SMD = −0.23, 95% CI: −0.51 to 0.04, *p* = 0.10) and 12 weeks (SMD = 0.21, 95% CI: −0.41 to 0.83, *p* = 0.51). For mindfulness, there were no significant differences in SMD between the two subgroups: 8 weeks (9, 33, 37), 16 weeks (38) (*p* = 0.69). MBIs had significant effects among the study with 16 weeks (SMD = 0.51, 95% CI: 0.15 to 0.88, *p* = 0.006). However, no significant intervention effect was found for 8 weeks (SMD = 0.34, 95% CI: −0.41 to 1.09, *p* = 0.37).

### 3.6. Publication bias

It was not possible to test for publication bias by drawing funnel plots due to the inclusion of <10 articles in the single meta-analysis, suggesting that potential publication bias may exist in this study.

## 4. Discussion

### 4.1. Discussion of pooled results

The results showed that the MBIs significantly reduced stress scores and produced high improvements (SMD = −0.84), but they had no significant effect on anxiety, depression, or mindfulness scores in menopausal women compared to the control group. It is important to note that the results should be treated with caution due to the statistical heterogeneity in the study. This study investigated the effect of each study on overall risk by using sensitivity analysis to explore the main sources of heterogeneity. Large differences in a sample size (range: 27–197), different intervention types, weekly intervention hours (range: 30–300 min/week), intervention duration (range: 8–16 weeks), control group type (e.g., wait-list, routine health care, etc.), measurement instruments, cultural background, or other confounding factors may be responsible for heterogeneity.

Although the exact mechanism of MBIs for menopausal women is currently unclear, some arguments have been made through research that MBIs can cultivate people to keep an open mind and an observational attitude, improve reaction flexibility and emotional tolerance (47), interrupt rumination on past experiences and worry about future events (48), and then improve negative emotions and cope with stress (16, 49). This helps a person more effectively decide how to respond to mental, emotional, or behavioral problems (50–52). The neurobiological mechanisms involved suggest that stress-related hormones (e.g., cortisol) negatively affect emotions by increasing the volume of the amygadala nucleus and decreasing the volume of the prefrontal cortex and hippocampus (53). In contrast, MBIs reduce the volume of the amygdala nucleus and increase the volume of the hippocampus (54, 55). Studies have shown that estrogen inhibits sympathetic activity and enhances parasympathetic activity (56, 57). Lower estrogen levels during menopause lead to increased sympathetic activity and inhibition of baroreceptors (58–60). MBIs improve body awareness and self-regulation by balancing sympathetic and parasympathetic responses and decreasing hypothalamic-pituitary-adrenal activation (61); It also increases stress-related autonomic activation (61), causes the brain to make new responses, and reorganizes neural pathways, which could change the structure of the brain in the long run (62). The stress-attenuating effects of MBIs work by reducing stress reactivity and activation (63), as well as by changing psychobiological stress markers like cortisol, C-reactive protein, and triglycerides (64). This has a long-term effect on buffering the stress response (65). Through this process, it allows individuals to better adapt to their environment (66–68), which contributes to alleviating the anxiety, depression, and stress experienced by women as they face menopause (69). In terms of application, MBIs have been widely used. Many studies have shown positive effects of MBIs in relieving anxiety and depression (49, 70), reducing stress (71, 72), and increasing mindfulness (73, 74).

In this study, MBIs had no significant effect on anxiety, depression, or mindfulness scores in menopausal women, which is related to the missing rate due to poor participant compliance. According to Nurdilan et al. (38), the missing rate was > 20%, which has affected other participants' motivation to maintain the MBIs (75). Further subgroup analysis revealed that the effects of MBIs may depend on the continent and the length of the intervention (weeks). There were cross-cultural differences in the intervention effects of MBIs on anxiety. The effectiveness of the intervention is significantly higher in Asia than in Europe, which stems from differences in the conceptual understanding of mindfulness between East and West. Mindfulness refers more to a state of being in the present moment, which is essentially equivalent to “Vipassana” in Eastern Buddhism (76). The difference in the understanding of mindfulness between Eastern and Western due to different ideologies and political systems leads to a gap in the level of mindfulness (77); since depression and mindfulness are influenced by multiple factors, mindfulness as a moderating variable is difficult to change in a short period of time (78). It has also been shown that although MBIs can cultivate people's better insight and self-regulation abilities, they are also regarded as an idealized state in cognitive science theory and are difficult to achieve in a short period of time (79). Further high-quality studies with large samples are needed to explore the effectiveness of MBIs in menopausal women.

However, the results of this study showed no statistically significant differences between the experimental and control groups in anxiety, depression, and mindfulness scores and only statistically significant differences in stress scores. Although the results were not statistically significant, the findings also inform the development of MBIs for menopausal women. Further studies are still needed to validate these results and follow the long-term effects.

### 4.2. Limitations

This meta-analysis is the first to definitively show that MBIs can significantly reduce stress scores in menopausal women, but they have no significant effect on anxiety, depression, and mindfulness scores. However, there are some limitations in this study: (1) Only eight studies detailed the randomization method due to the study design limitations. While the other studies only mentioned randomization without specifying the method being used, and only four studies implemented blinding; (2) The difficulty of conducting more subgroup analyses under the limitation of the number of included studies may lead to some heterogeneity among studies; (3) Potential regulatory variables, such as subject characteristics, intervention types, control group types, missing rates, and other factors, may have varying degrees of influence on the results. This study did not conduct a stratified analysis of potential influencing factors due to the limitations of the study design. (4) The possibility of publication bias cannot be ruled out because the number of included studies limited the ability to detect publication bias; (5) There was no uniform measurement instrument for the same outcome index. Although SMD was chosen as the effect size indicator, caution is needed when interpreting the results.

### 4.3. Implications for practice and research

The findings have important implications for clinical practice, as the adoption of MBIs can be effective in reducing stress in menopausal women. First, further research should be conducted in the future on how to increase participants' motivation, reduce the missing rate, and maintain the effects of MBIs; second, it was not possible to conduct subgroup analyses for the types of interventions due to the limitations of the studies included in this study. As a result, future studies should conduct stratified, in-depth comparisons and discussions of various types of interventions. In the future, researchers will need to do more high-quality studies with larger sample sizes to confirm that MBIs work for menopausal women.

## 5. Conclusion

The results of this study revealed that MBIs significantly reduced the stress scores of menopausal women but did not significantly improve their anxiety, depression, and mindfulness scores. The effectiveness of the MBIs on anxiety, depression, and mindfulness scores in menopausal women needs to be further validated in future studies with large, high-quality samples.

## Data availability statement

The original contributions presented in the study are included in the article/Supplementary material, further inquiries can be directed to the corresponding author.

## Author contributions

HL and HZ: conceptualization and methodology. HL: software, validation, formal analysis, and writing—original draft preparation. HZ: reviewing, editing, and supervision. HL, JW, and KC: resources and data curation. All authors contributed to the article and approved the submitted version.
