# Supplementary material for: The effects of mindfulness-based interventions on anxiety, depression, stress, and mindfulness in menopausal women: A systematic review and meta-analysis
Source: Front Public Health. 2023 Jan 9;10:1045642. doi: 10.3389/fpubh.2022.1045642 (PMC9869042; doi:10.3389/fpubh.2022.1045642)
Supplement: Supplementary file 1 [file Table_1.DOCX]

Supplementary Material

# Search strategy

| Database | Search Terms |
| --- | --- |
| Pubmed | #1. "Mindfulness"[MeSH Terms] OR "Meditation"[MeSH Terms] OR "Mindfulness"[Title/Abstract] OR "Meditation"[Title/Abstract] OR "Mindfulness meditation"[Title/Abstract] OR "mindfulness-based intervention"[Title/Abstract] OR "MBSR"[Title/Abstract] OR "mindfulness-based stress reduction"[Title/Abstract] OR "mindfulness-based cognitive therapy"[Title/Abstract] OR "Vipassana"[Title/Abstract] OR "Mindfulness training"[Title/Abstract]  #2. "perimenopause"[MeSH Terms] OR "Menopause"[MeSH Terms] OR "Menopause"[Title/Abstract] OR "Menopausal transition"[Title/Abstract] OR "menopausal syndrome"[Title/Abstract] OR "Peri"[Title/Abstract] OR "perimenopause"[Title/Abstract] OR "Perimenopause period"[Title/Abstract] OR "Perimenopausal"[Title/Abstract] OR "Perimenopausal period"[Title/Abstract] OR "Perimenopausal transition"[Title/Abstract] OR "perimenopausal syndrome"[Title/Abstract] OR "climacteric"[MeSH Terms] OR "climacteric syndrome"[Title/Abstract] OR "Climacterium"[Title/Abstract]  #3. #1 AND #2 |
| Cochrane Library | #1. MeSH descriptor: [Mindfulness] explode all trees  #2. MeSH descriptor: [Meditation] explode all trees  #3. (“mindfulness”):ti,ab,kw OR (“meditation”):ti,ab,kw OR (“Mindfulness meditation”):ti,ab,kw OR (“mindfulness-based intervention”):ti,ab,kw OR (“MBSR”):ti,ab,kw  #4. (“mindfulness-based stress reduction”):ti,ab,kw OR (“mindfulness-based cognitive therapy”):ti,ab,kw OR (“Vipassana”):ti,ab,kw OR (“Mindfulness training”):ti,ab,kw  #5. #1 OR #2 OR #3 OR #4  #6. MeSH descriptor: [Perimenopause] explode all trees  #7. MeSH descriptor: [Menopause] explode all trees  #8. MeSH descriptor: [Climacteric] explode all trees  #9. (“menopause”):ti,ab,kw OR (“Menopausal transition”):ti,ab,kw OR (“menopausal syndrome”):ti,ab,kw OR (“Peri”):ti,ab,kw OR (“perimenopause”):ti,ab,kw  #10. (“Perimenopause period”):ti,ab,kw OR (“Perimenopausal”):ti,ab,kw OR (“Perimenopausal period”):ti,ab,kw OR (“Perimenopausal transition”):ti,ab,kw OR (“perimenopausal syndrome”):ti,ab,kw  #11. (“climacteric syndrome”):ti,ab,kw OR (“Climacterium”):ti,ab,kw  #12. #6 OR #7 OR #8 OR #9 OR #10 OR #11  #13. #5 AND #12 |
| Embase | #1. 'mindfulness'/exp  #2. 'meditation'/exp  #3. mindfulness:ti,ab,kw OR meditation:ti,ab,kw OR 'mindfulness meditation':ti,ab,kw OR 'mindfulness-based intervention':ti,ab,kw OR mbsr:ti,ab,kw OR 'mindfulness-based stress reduction':ti,ab,kw OR 'mindfulness-based cognitive therapy':ti,ab,kw OR vipassana:ti,ab,kw OR 'mindfulness training':ti,ab,kw  #4. #1 OR #2 OR #3  #5. 'climacterium'/exp  #6. 'menopause'/exp  #7. menopause:ti,ab,kw OR 'menopausal transition':ti,ab,kw OR 'menopausal syndrome':ti,ab,kw OR peri:ti,ab,kw OR perimenopause:ti,ab,kw  #8. #5 OR #6 OR #7  #9. #4 AND #8 |
| Web of Science | #1. TS=("Mindfulness") OR TS=("Meditation") OR TS=("Mindfulness meditation") OR TS=("mindfulness-based intervention") OR TS=("MBSR") OR TS=("mindfulness-based stress reduction") OR TS=("mindfulness-based cognitive therapy") OR TS=("Vipassana") OR TS=("Mindfulness training")  #2. TS=("Peri") OR TS=("perimenopause") OR TS=("Perimenopause period") OR TS=("Perimenopausal") OR TS=("Perimenopausal period") OR TS=("Perimenopausal transition") OR TS=("perimenopausal syndrome") OR TS=("Menopause") OR TS=("Menopausal transition") OR TS=("menopausal syndrome") OR TS=("Climacteric") OR TS=("climacteric syndrome") OR TS=("Climacterium")  #3. #1 AND #2 |
| CNKI  (China National Knowledge Infrastructure) | #1. (主题=正念 OR 正念疗法 OR 正念减压 OR 正念认知 OR 正念干预 OR 正念训练 OR 正念冥想 OR正念减压疗法 OR 正念认知疗法)  #2. (主题=围绝经期 OR 更年期 OR 更年期综合征 OR 围绝经期综合征)  #3. #1 AND #2 |
| Wanfang | #1. 主题:(正念 OR 正念疗法 OR 正念减压 OR 正念认知 OR 正念干预 OR 正念训练 OR 正念冥想 OR正念减压疗法 OR 正念认知疗法)  #2. 主题:(围绝经期 OR 更年期 OR 更年期综合征 OR 围绝经期综合征)  #3. #1 AND #2 |
